# Mechanisms of Self-Sustained Oscillatory States in Hierarchical Modular Networks with Mixtures of Electrophysiological Cell Types

**DOI:** 10.3389/fncom.2016.00023

**Published:** 2016-03-23

**Authors:** Petar Tomov, Rodrigo F. O. Pena, Antonio C. Roque, Michael A. Zaks

**Affiliations:** ^1^Institute of Mathematics, Humboldt University of BerlinBerlin, Germany; ^2^Laboratório de Sistemas Neurais, Department of Physics, School of Philosophy, Sciences and Letters of Ribeirão Preto, University of São PauloSão Paulo, Brazil; ^3^Institute of Physics, Humboldt University of BerlinBerlin, Germany; ^4^Institute of Physics and Astronomy, University of PotsdamPotsdam, Germany

**Keywords:** self-sustained activity, cortical oscillations, irregular firing activity, hierarchical modular networks, cortical network models, intrinsic neuronal diversity, up-down states, chaotic neural dynamics

## Abstract

In a network with a mixture of different electrophysiological types of neurons linked by excitatory and inhibitory connections, temporal evolution leads through repeated epochs of intensive global activity separated by intervals with low activity level. This behavior mimics “up” and “down” states, experimentally observed in cortical tissues in absence of external stimuli. We interpret global dynamical features in terms of individual dynamics of the neurons. In particular, we observe that the crucial role both in interruption and in resumption of global activity is played by distributions of the membrane recovery variable within the network. We also demonstrate that the behavior of neurons is more influenced by their presynaptic environment in the network than by their formal types, assigned in accordance with their response to constant current.

## 1. Introduction

Networks of cortical neurons display sustained activity even in the absence of external input. Evidence of this self-sustained activity (SSA) comes from *in vitro* preparations of cortical tissue slices (Sanchez-Vives and McCormick, 2000; Mao et al., 2001; Cossart et al., 2003; Shu et al., 2003) or cell cultures (Plenz and Aertsen, 1996; Wagenaar et al., 2006), and *in vivo* cortical “slab” preparations (Burns and Webb, 1979; Timofeev et al., 2000; Lemieux et al., 2014). Sustained cortical activity is also observed in situations in which the brain is essentially disconnected from external stimuli, as in slow-wave sleep (SWS) and anesthesia (Steriade et al., 1993; Contreras and Steriade, 1995; Steriade et al., 2001).

Electrophysiological studies have shown that SSA states in the above situations share the same basic features (Sanchez-Vives and McCormick, 2000; Mao et al., 2001; Steriade et al., 2001; Cossart et al., 2003; Shu et al., 2003; Kaufman et al., 2014). As revealed by EEG or local field potential measurements, they are characterized by slow (< 1 Hz) network oscillations, consisting of epochs of high network activity intercalated with periods of nearly absent network activity. There is a close correspondence between this slow network oscillation and the underlying behavior of single network neurons (as revealed by intracellular measurements). During near quiescent network activity single neurons have hyperpolarized membrane potentials close to resting state (down state) and during high network activity single neurons have depolarized membrane potentials close to firing threshold (up state).

Some hypotheses have been put forward to explain how self-sustained up and down states can originate and be maintained in cortical networks, among them synaptic noise (Timofeev et al., 2000; Holcman and Tsodyks, 2006; Parga and Abbott, 2007), spontaneously firing neurons (Compte et al., 2003; Hill and Tononi, 2005) and an interplay between two cortical layers, one of them displaying SSA and the other displaying transient activity (Destexhe, 2009). However, the specific mechanisms that might implement them are still subject of experimental investigation and no definite conclusion could be reached yet (Compte, 2006; Mann et al., 2009; Chauvette et al., 2010; Destexhe and Contreras, 2011; Harris and Thiele, 2011).

Recently, we reported the emergence of SSA states in a computational model of the cortex with hierarchical and modular architecture composed of neurons of different intrinsic firing behaviors (Tomov et al., 2014). The neurons belonged to the five main electrophysiological cell classes found in the cortex (Connors et al., 1982; McCormick et al., 1985; Nowak et al., 2003; Contreras, 2004): the excitatory regular spiking, chattering and intrinsically bursting neurons, and the inhibitory fast spiking and low threshold spiking neurons. In the regions of the parameter space where the inhibitory synaptic strength exceeds the excitatory synaptic one, i.e., the regions where there is a balance between excitation and inhibition (Shadlen and Newsome, 1994; van Vreeswijk and Sompolinsky, 1996; Amit and Brunel, 1997; van Vreeswijk and Sompolinsky, 1998), we observed SSA states with spiking characteristics similar to the ones observed experimentally. The detected SSA states were transiently chaotic, possessed finite lifetimes, and displayed large-scale network activity oscillations with alternating high and low global-activity epochs followed by abrupt unpredictable decay toward the resting state. The lifetime expectancy depended on network modularity, on mixture of neurons of different types, and on excitatory and inhibitory synaptic strengths. For fixed network parameters the lifetimes of the transient SSA states obeyed exponential distributions.

Remarkably, the states with longest lifetime expectations in the region of parameter space with physiologically plausible mean network firing rates displayed collective oscillatory behavior that resembled self-sustained up and down states found in cortical networks. Global frequencies generated by our network models (~ 4 − 7 Hz) were higher than the slow frequencies (< 1 Hz) observed both *in vitro* and *in vivo*, that may be due to factors like network size, absence of time delays in the network connections or proper adjustment of synaptic time constants. However, the very presence of these sustained collective oscillations in the broad parameter range demands an investigation of the mechanisms responsible for their origin and characteristics.

Below, we analyze the properties of the network model and explain our previous observations. To understand the origin of the exponential distribution of the SSA lifetimes, we develop a phenomenological global description of this transiently chaotic state and discuss the importance of mixture of different neuronal types in the network. In the framework of this description we see that the presence of modular structures in the network architecture facilitates the sustainment of activity. Further, we relate the mechanisms of rise and fall of global activity to properties of individual neurons of the network. Within our model, each neuron is characterized by two variables: voltage and recovery. Fixing the parameter combination that ensures sufficiently long SSA, we find out that the onset and temporary cessation of activity in the ensemble of neurons are governed by instantaneous distributions of the recovery variable among its members. This effect is explained on the basis of the single neuron dynamics. Proceeding to the ensemble we see that embedding into a network is able to change qualitatively the spiking patterns of the units, so that they do not conform anymore to the classification based on the single neuron dynamics.

Therefore, by dissecting the model we are able to determine network and intrinsic neuronal mechanisms responsible for the self-sustained oscillatory (up and down) behavior observed in the simulations. This provides a way to bridge between network and neuronal activity states and how they influence each other.

## 2. Methods

### 2.1. Network: construction and measures

#### 2.1.1. Modular architecture

Our studies are focused on activity in neuronal networks that, mimicking the cortical network at meso- and macroscopic scales, possess hierarchical modular structure. Since inhibitory couplings are known to be typically present only in the short-range connections, whereas excitatory couplings are detected both in local and in long-range connections (Binzegger et al., 2004; Voges et al., 2010), we demand that all intermodular links are excitatory. Besides, in accordance with anatomical evidence (Boucsein et al., 2011), there should be more connections between close modules than between faraway ones.

The hierarchical modular network is generated by the following top-down algorithm (Kaiser and Hilgetag, 2010; Wang et al., 2011): We start from randomly connected *N* = 2^10^ neurons, with the physiologically motivated ratio of excitatory to inhibitory neurons 4:1; for every pair of randomly chosen neurons *i* and *j*, the probability of connection *i* → *j* is 0.01. Thereby, separate connection probabilities are, respectively, *p*_*e*_ = 0.008 for excitatory connections and *p*_*i*_ = 0.002 for inhibitory ones. We assign to this network the hierarchical level *H* = 0.

At the next step, we randomly divide all neurons into two modules of equal size. All connections *within* the modules are preserved. Since inhibitory connections *between* the modules are not allowed, all such links are rewired: cut (detached from postsynaptic neurons) and redirected back into the modules of their presynaptic neurons, where they are attached randomly. The fate of each excitatory link between the modules is decided at random: with probability *p*_*r*_ = 0.1 a connection is retained, and with probability (1 − *p*_*r*_) = 0.9 it is cut and rewired back into the module with the presynaptic neuron. In this way we obtain two modules, sparsely interconnected by excitatory links, and assign to this network the level *H* = 1. Although the total numbers of excitatory and inhibitory connections are preserved during this procedure, rewiring makes the connectivity inhomogeneous: Inside each module the density of inhibitory connections is doubled. The density of excitatory connections inside the modules is increased by the factor 2-*p*_*r*_, whereas the density of excitatory connections between the modules is decreased and becomes *p*_*e*_*p*_*r*_.

Repeating the same procedure: halving the existing modules and rewiring the intermodular links, so that all inhibitory connections and 90% of the excitatory connections between the modules are rewired back, we obtain networks with *H* = 2,3…; a network with hierarchical level *H* has 2^*H*^ modules.

**Figure 1:**
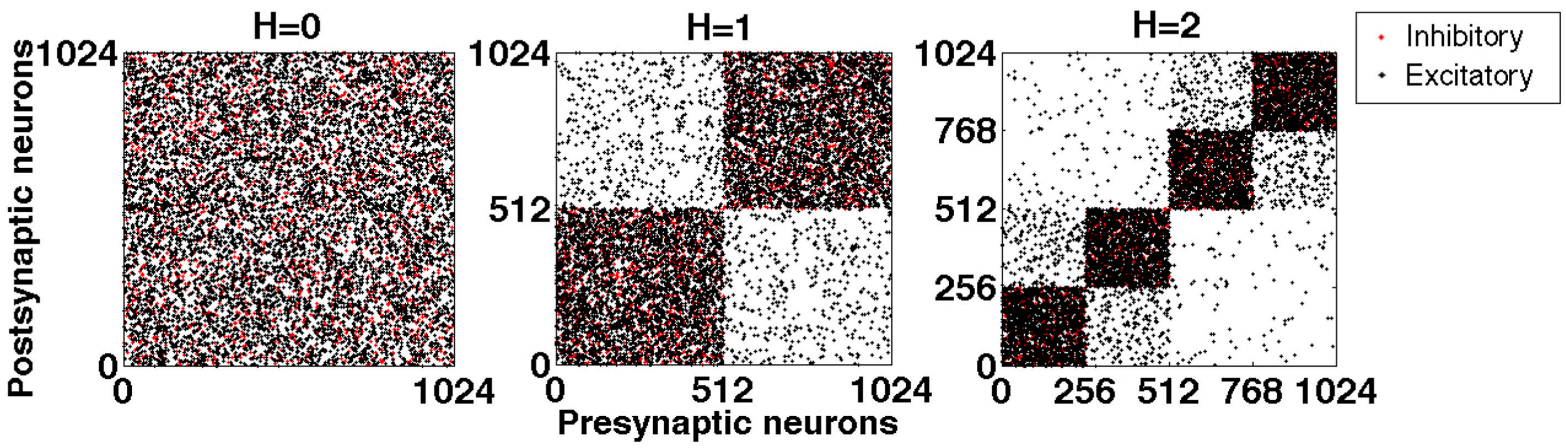
**Exemplary connection matrices for networks with hierarchical levels *H* = 0, 1, and 2, constructed by the top-down procedure**. Black dots: excitatory connections. Red dots: inhibitory connections. Note that the latter never interconnect different modules.

Figure 1 shows exemplary networks generated by this algorithm for hierarchical levels *H* = 0, 1 and 2.

If during the procedure a pair of modules has been obtained by halving a larger one, we say that these two modules are “hierarchically close”; otherwise they are “hierarchically distant.” In the network of hierarchical level *H* = 2 shown in the right panel of **Figure 5**, for each of the four modules there are one close and two distant ones; the hierarchically close pairs are (1 and 2) and (3 and 4). Hierarchical distance has explicit quantitative meaning: Due to the procedure of cutting and rewiring, the number of excitatory connections between the close modules is almost twice (by the factor 1.9) higher than between the distant ones. At the same time, this procedure rises the level of inhibition in the modules: The density of inhibitory connections inside the modules at *H* = 2 is 4 times higher than it was at *H* = 0: in the latter case a chosen neuron could obtain input from any inhibitory neuron in the network with probability *p*_*i*_; at *H* = 2 only inhibitory neurons from the same module matter, but for each of them the probability of being presynaptic to the chosen neuron is 4*p*_*i*_.

### 2.2. Network measures

Here, we introduce measures that we use below for evaluation of the network characteristics. For the neuron *i*, the spike train is represented as a series of δ functions, $x_{i}\left(t\right)=\underset{\left\{t_{i}^{f}\right\}}{\sum }\delta \left(t-t_{i}^{f}\right)$ with $\left\{t_{i}^{f}\right\}$ being the set of time instants at which the neuron *i* fires a spike. The mean firing rate for a set of *N* neurons over a time interval *T* is defined as

$$\begin{array}{l} \langle f\rangle =\frac{1}{N}\sum_{i\;=\;1}^{N}\frac{1}{T}\int_{T}x_{i}(t\prime )dt\prime , \end{array}$$

and the time-dependent firing rate for the same set as

$$\begin{array}{l} f(t;\Delta t)=\frac{1}{N}\sum_{i\;=\;1}^{N}\int_{t}^{t+\Delta t}x_{i}(t\prime )dt\prime . \end{array}$$

Below, we fix Δ*t* at 0.01 ms and shorten *f*(*t*; Δ*t*) to *f*(*t*). To activate the network, we apply within limited time intervals stimulating external current to various groups of neurons. When the stimulation is switched off, the time count is reset at zero. The lifetime of a neuron *i* is defined as the time of the last spike in its spike train:

$$\begin{array}{l} t_{i}^{\text{last}}=\max\{t_{i}^{f}\}, \end{array}$$

and the network lifetime is the longest lifetime among all neurons: $L\equiv \max\left\{t_{i}^{\text{last}}:i=1,..,N\right\}.$

While analyzing the oscillations, we divide each record into different epochs according to the following procedure: First, for each module *j* in the network, we determine its maximal firing rate over the duration of the entire simulation, *M*_*j*_ = max{*f*_*j*_(*t*)}, and identify the start (end) of high activity as the time value, at which the firing rate *f*(*t*) in that module exceeds (falls below) 5% of *M*_*j*_.

In terms of the entire network, we define, respectively, the beginning of high global activity as its start in *any* module, and the end as the moment when the activity ceases in *every* module. In this way, we put four markers at different stages of network activity: the beginning, the midpoint and the end of each epoch of high global activity, and the middle of an epoch of low activity. Absence of high activity is interpreted as low activity (unless the system is not in its stable state of rest in which all activity is terminated, and which cannot be left without external stimulation). The middle of the epoch of low activity is, therefore, defined as the midpoint between the end of the previous epoch of high activity and the beginning of the next one.

Further, we will need to characterize distributions of individual neuronal variables within the network. For the recovery variable *u* (see below) the distribution U(*u*, δ*u*; *t*) at time *t* is defined as

(1) $$\begin{array}{l} U(u,\delta u;t)=\frac{1}{N}\sum_{i=1}^{N}\int_{u}^{u+\delta u}\delta (u_{i}(t)-x)dx. \end{array}$$

where summation is performed over the whole network.

### 2.3. Neuron models

Having generated a network, we need to populate each of its sites by a functioning neuron. For that purpose we use the model proposed in Izhikevich (2003). Its detailed analysis can be found in Izhikevich (2007); here, we review the basic features, relevant for our studies. Within this approach, the firing behavior of a single neuron is characterized by two variables: membrane voltage *v* and recovery variable *u*, that at *v* < *v*_peak_ = 30*mV* obey the coupled differential equations

(2) $$\begin{array}{l} \dot{v}=\underset{}{\underset{︸}{0.04v^{2}+5v+140}}-u+I(t)\\ \;=\text{ f}(v)\;-u+I(t)\\ \dot{u}=a(bv-u), \end{array}$$

and at the threshold *v*(*t*) = *v*_peak_ the variables are instantaneously reset according to the rule

(3) $$\begin{array}{l} v(t)\mapsto c,\;u(t)\mapsto u(t)+d. \end{array}$$

Depending on the values of the four parameters (*a*, *b*, *c*, *d*), the firing pattern of the model neuron belongs to one of the five electrophysiological classes used in the simulations: the regular spiking (RS), intrinsically bursting (IB), and chattering (CH) neurons are excitatory, whereas the fast spiking (FS) and low-threshold spiking (LTS) ones are inhibitory (see Table 1). Notation describes characteristic firing patterns at constant input: of the five types, only the CH and IB neurons are capable to fire patches (bursts) of spikes: all other neurons issue sequences of solitary spikes.

**Table 1:** **Parameters (*a, b, c, d*) characterizing five electrophysiological cell classes used in the simulations**.

	****a****	****b****	****c****	****d****
RS	0.02	0.2	−65	8
CH	0.02	0.2	−50	2
IB	0.02	0.2	−55	4
FS	0.1	0.2	−65	2
LTS	0.02	0.25	−65	2

For a neuron embedded in a network, the synaptic input current varies in time. We model *I*(*t*) as

(4) $$\begin{array}{l} I_{\text{syn}}(t)=G_{\text{ex}}(t)(E_{\text{ex}}-v)+G_{\text{in}}(t)(E_{\text{in}}-v), \end{array}$$

where *G*_ex_(*t*) is the total conductance of all excitatory synapses of the neuron, *G*_in_(*t*) is the total conductance of its inhibitory synapses, *E*_ex_ and *E*_in_ are the reversal potentials of, respectively, excitatory and inhibitory synapses.

Whenever a presynaptic excitatory or inhibitory neuron fires, the total excitatory or inhibitory conductances *G*_ex/in_(*t*) are increased by fixed increments *g*_ex/in_. Otherwise, they decay exponentially with time constants τ_ex/in_:

(5) $$\frac{d}{dt}G_{\text{ex/in}}(t)=-\frac{G_{\text{ex/in}}(t)}{\tau_{\text{ex/in}}}+g_{\text{ex/in}}\sum_{\begin{array}{c} j\in \text{presyn}\\ t_{j}^{f}<t \end{array}}\delta (t-t_{j}^{f}),$$

where $t_{j}^{f}$ denotes the firing time of a presynaptic neuron j.

The values *E*_ex_ = 0 mV, *E*_in_ = −80 mV, τ_ex_ = 5 ms and τ_in_ = 6 ms, that we employ in Equations (4) and (5), are representative of AMPA and GABA*_A_* receptor-mediated excitatory and inhibitory synapses, respectively. Synaptic delays are neglected. Throughout the whole paper we consider a single pair *g*_ex_ = 0.15, *g*_in_ = 1.0 of increments of the synaptic conductances: at these values, oscillatory self-sustained activity was steadily reproduced in the studied network (Tomov et al., 2014).

For comparison, we present a few computational results for a network that features the same topology but obeys a different dynamical system for each neuron: the AdEx (adaptive exponential integrate and fire) model (Brette and Gerstner, 2005), governed by the equations:

(6) $$\begin{array}{l} C\dot{v}=\underset{}{\underset{︸}{-g_{L}(v-E_{L})+g_{L}\Delta_{T}\exp(\frac{v-v_{T}}{\Delta_{T}})}}-u+I(t)\\ \text{ f}(v)\\ \tau_{u}\dot{u}=a(v-E_{L})-u, \end{array}$$

where at the threshold *v*(*t*) = *v*_T_, the variables are instantaneously reset:

(7) $$v(t)\mapsto v_{r},\;u(t)\mapsto u(t)+b.$$

The choice of parameters is based on (Naud et al., 2008; Destexhe, 2009) with few modifications: all neurons share *C* = 200, *E*_*L*_ = −70, Δ_*T*_ = 2, *v*_*T*_ = −30, a = 2; τ_*u*_ = 200, *v*_*r*_ = −60; excitatory neurons have *g*_*L*_ = 12 and *b* = 300 whereas inhibitory neurons have *g*_*L*_ = 10 and *b* = 0.

Synaptic inputs to the AdEx neurons are modeled by Equations (4) and (5); the increments *g*_ex/in_ used for the network with AdEx neurons are 15 and 70, respectively.

### 2.4. Ensemble of trajectories

We intend to obtain an adequate and sufficiently informative picture of the oscillatory SSA: an apparently irregular time-dependent process that lacks periodicity and looks chaotic. Numerical experiments confirm that minor changes in initial conditions for just a few neurons may not only change the order in which the neurons enter and leave the active state, but also strongly affect the duration of the SSA as a whole.

Sensitivity to minute details of initial state does not allow to draw far-reaching conclusions from properties of single numerical trajectories. Exhaustive analysis in the 3*N*-dimensional phase space of the discontinuous dynamical system at *N* = 2^10^ seems hardly feasible. However, statistical descriptions, based on distributions in a sufficiently large ensemble of networks that share the topology but differ by their preparation (duration and intensity of initial stimulation, location of stimulated neurons in the network, etc.) prove to be quite robust.

For preparation of ensembles we do not choose initial conditions at random in the high-dimensional phase space of the network: a representative sampling would be hardly available computationally. Instead, we start at the state of rest, and stimulate randomly taken groups of neurons with constant external current. After the onset of global activity in the network, we switch the stimulation off and let the network evolve freely. This procedure results in a sort of “importance sampling”: it leads the system to initial positions, that are close to typical pathways in the “physiologically reasonable” part of its phase space, i.e., presumably close to segments of long-living SSA trajectories. We create ensembles of such initial conditions by varying

- the proportion of randomly chosen stimulated neurons: 1, 1/2, 1/8, 1/16;
- the amplitude of the external current from *I*_stim_ = 10 to *I*_stim_ = 20; and
- the duration of stimulation from 50 to 300 ms.

In the latter case, the range of variation by far exceeds the typical interval of approximately 100 ms between two consecutive epochs of global activity in the network. These measures enable us to obtain ensembles of trajectories with robust, reproducible statistical characteristics.

### 2.5. Auxiliary ensemble along the reference trajectory

Ensembles of trajectories created with the help of the above procedure yield global characteristics of dynamics but say only a little about the local structure of the phase space in the neighborhood of the chaotic set. To resolve this structure, we create an auxiliary ensemble in the following way.

1. At fixed parameter values we take from the ensemble a single orbit with SSA lifetime larger than 2000 ms (over 20 subsequent epochs of high global activity); this ensures that the orbit stays close to the chaotic set sufficiently long. Below, we call this orbit a *reference trajectory* R(*t*).
2. On R(*t*), we choose fifty equidistant positions P*_k_*, *k* = 1, .., 50 at the time values
  (8) $$\begin{array}{l} P_{k}=R(t_{0}+k\Delta t) \end{array}$$
  with *t*_0_ = 370 ms and Δ*t* = 7 ms. The offset ensures that by the time *t*_0_ the reference trajectory has reached the region of the chaotic set (this has been established visually from the irregular shape of oscillations). The choice of Δ*t* enables us to have within each epoch of global activity and subsequent inactivity ≈15 positions P*_k_*: a reasonably dense covering of R(*t*).
3. In each position the system is perturbed six hundred times, by stimulating for 3 ms each eighth neuron with the external input current *I*_ext_ = 10. For every perturbation the 128 stimulated neurons are chosen at random. The stimulation interval is much shorter than the characteristic time of the system ≈ 100 ms, therefore within it the perturbed orbits stay sufficiently close to the reference trajectory.
4. After the perturbation the system is left to evolve freely, and the resulting lifetime is recorded.

This local procedure creates 50 sets of “secondary” initial conditions, each one with 600 points close to one of P*_k_*. Since, by construction, all these sets lie at different locations along the reference trajectory, they provide information about local dynamics near the chaotic state, and, in particular, on the rates of escape from it.

**Figure 2:**
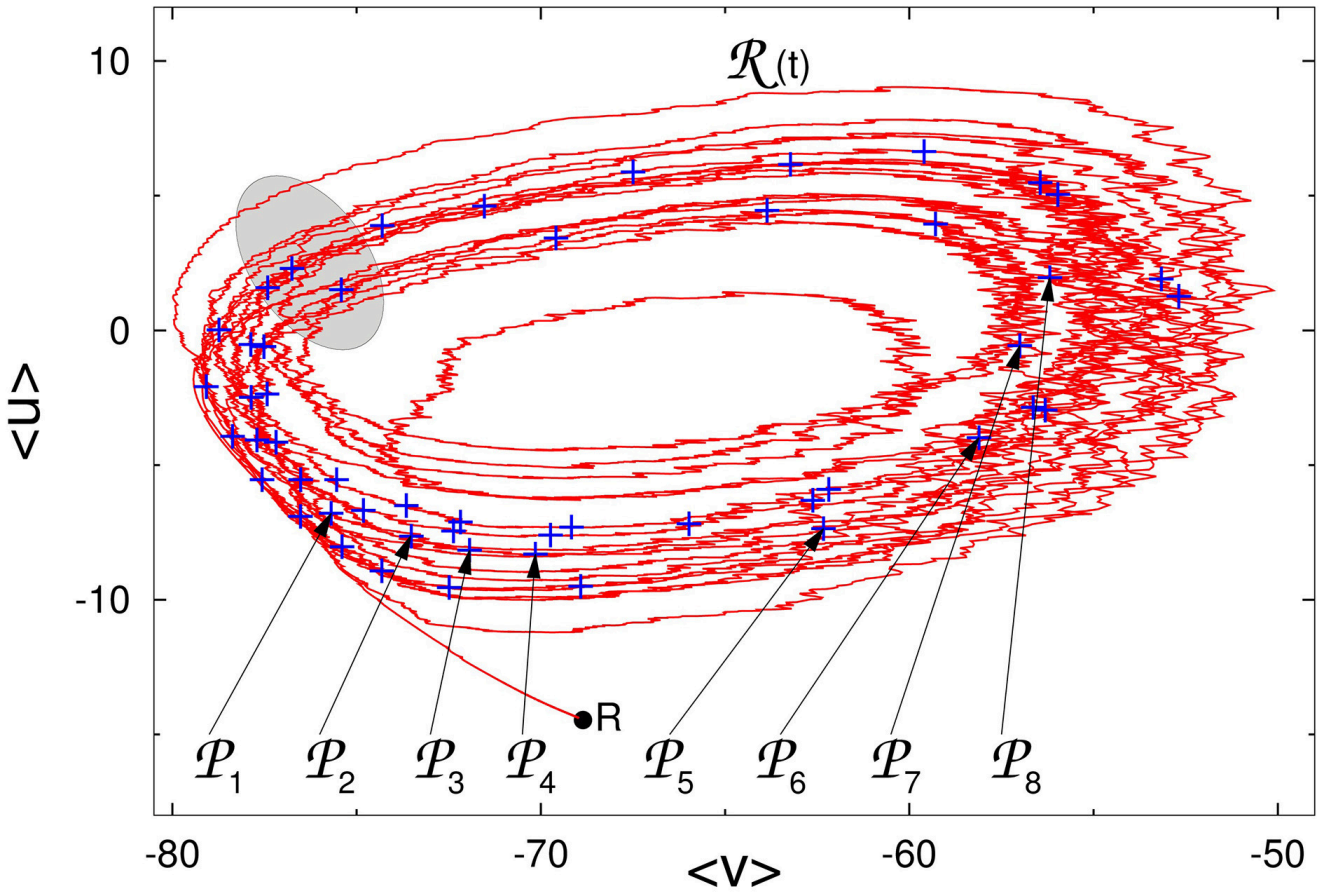
**Reference trajectory R(*t*) on the phase plane of averaged values of voltage and membrane recovery variable**. Pluses: positions of perturbations P*_k_* along R(*t*). Gray region: approximate location of the “hole” (see explanations in the text). *R*: state of rest.

In Figure 2 we show the projection of the reference trajectory R(*t*) and positions of perturbations P*_k_* on the plane of two artificial collective coordinates 〈*v*〉 and 〈*u*〉: instantaneous mean values of, respectively, voltage and membrane recovery variable over all 2^10^ neurons.

For the numerical simulations of the network we have used the original C++ code. Data analysis has been conducted in Matlab.

## 3. Results

### 3.1. SSA: network perspective

#### 3.1.1. Preliminaries

In Tomov et al. (2014) we observed that in the broad range of values of increments of synaptic conductances, initial stimulation of the system led to repeated epochs of intense correlated activity, separated by time intervals in which the majority of the neurons did not fire at all or fired seldom. Dynamics was aperiodic; the interval between the starting points of consecutive epochs of activity was typically in the range from 100 to 110 ms. After a certain time, the process abruptly ended with complete termination of activity: the system found itself at the stable state of rest. Remarkably, the entire duration of the process, as well as the number of the observed epochs of high activity, were highly sensitive to initial conditions: a minor variation in e.g., the length or strength of the initial stimulation often replaced a process with only a few such epochs by a process with several dozens of alternating onsets and breakdowns of activity, or vice versa. There seemed to be no precursor of the forthcoming termination of SSA: the last epochs of global activity qualitatively differed from all preceding ones neither in amplitude, nor in duration. We conjectured that the observed oscillatory SSA states were transiently chaotic: that in the phase space of the system there was a non-attracting chaotic invariant set (*chaotic saddle*) of zero measure, and that the generic trajectories did not stay in the vicinity of that set forever, but, after a shorter or longer motion along it, left it for the state of rest.

#### 3.1.2. Phenomenology

We start with a random network of 2^10^ neurons. Of them, 20% are inhibitory LTS neurons and 80% are excitatory ones: a mixture of 80% regular spiking neurons and 20% chattering neurons. The network is not divided into modules: *H* = 0.

Results of simulations show that after the end of stimulation the network displays a series (from several to several hundreds) of alternating epochs of global activity and inactivity that, on the level of separate neurons, do not reproduce each other and seem completely irregular. The series is followed by abrupt relaxation to the state of rest: in fact, activity is *transiently self-sustained*. During the active phase, projections of trajectories in the phase space of the system remind typical examples of deterministic chaos. The trajectory in Figure 2 is reminiscent e.g., of the Rössler attractor (Rössler, 1976). However, conventional indicators of chaos like Lyapunov exponents and fractal dimensions of the chaotic set are hardly applicable here due, first, to the high order (> 3000 variables) of the dynamical system and, second, to the finite lifetime of individual trajectories, which in many cases is too short to gather sufficient statistics. Hence, we are forced to use indirect evidence for our conclusions on the character of dynamics: our judgments are based on distributions of the activity lifetimes in the network. The lifetime here and below is understood as the length of time interval between the end of stimulation and the firing of the very last spike anywhere in the network.

We observe that a minute change in initial conditions (a small variation in the duration or strength of the stimulation) typically results in a strong—sometimes by orders of magnitude—variation of the lifetime. Besides, it turns out that for the sufficiently large ensemble of initial conditions, the distribution of lifetimes is exponential: the number *n*(*T*) of systems with lifetime larger than *T* approximately obeys the dependence

(9) $$n(T)\sim {\text{e}}^{-\kappa T}$$

**Figure 3:**
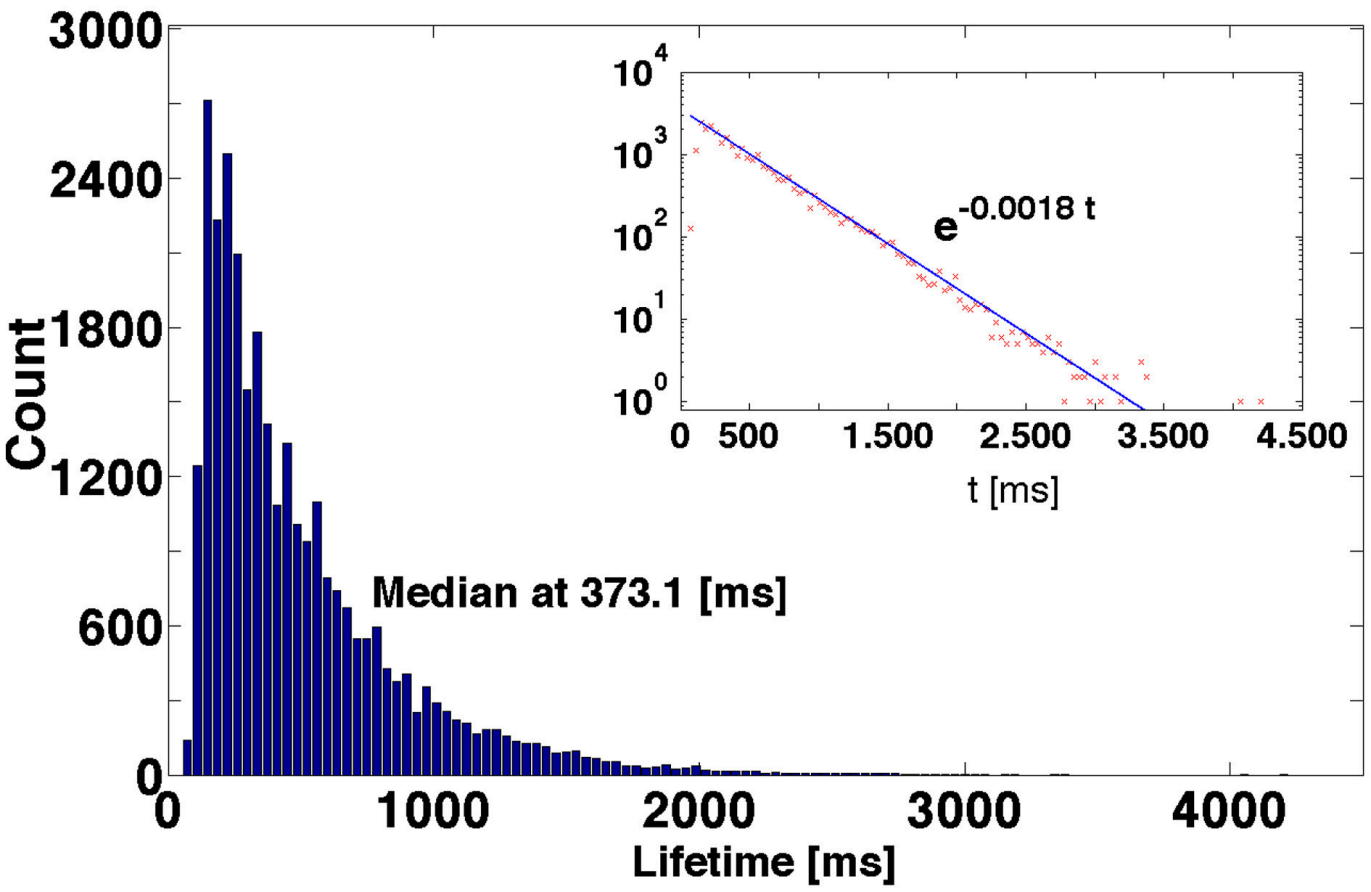
**Distribution of lifetimes of SSA for 3 × 10^4^ initial conditions obtained by perturbations of the reference trajectory**. Inset: Logarithmic representation of the ordinate.

A typical example is shown in Figure 3 the distribution of lifetimes for an ensemble of 3 × 10^4^ trajectories. For the non-attracting chaotic set, κ is the escape rate: In an ensemble of transiently chaotic trajectories, the value of $\tau_{\text{dec}}=\kappa^{-1}$ defines the characteristic time of decay of SSA in ms. If the network composition and the parameter values are fixed, the fitted value of κ, within numerical accuracy, appears to be the same for various sets of initial conditions; the prefactor and the exact shape of the curve may vary from a set to a set. Variation of parameters *g*_ex_ and *g*_in_ results in variation of the value of κ, but the exponential character of the distribution persists.

The effects observed: sensitive dependence of individual trajectories on initial conditions, and exponential distribution of lifetimes in the large ensemble of trajectories, are two characteristic attributes of the so-called transient chaos (Lai and Tél, 2011). Based on this, we conjecture that the oscillatory self-sustained activity in the network is transiently chaotic.

The escape rate κ is a global characteristics of the chaotic set. It does not tell whether the trajectories leave that set uniformly [like in case e.g., of radioactive decay, where in equal time intervals equal proportions of particles break up, and the number of survivors obeys the law (9)], or there are some preferential positions in the phase space for the termination of chaotic evolution. To find it out, we take a closer look at the local structure of the neighborhood of the chaotic set. For this purpose, we fix the parameter values *g*_ex_ = 0.15 and *g*_in_ = 1, choose a sufficiently long realization of SSA which we call the reference trajectory R(*t*), and create the auxiliary ensemble of trajectories near R(*t*) with the help of the procedure introduced in Methods (see Section 2.5).

If vicinities of all segments of the reference trajectory would offer the same possibility of immediate escape to the state of rest, the lifetimes of trajectories originating from different local sets would be comparable. Our numerical data unambiguously state that this is not the case. Rather, they indicate that escape occurs only from a relatively small local region responsible for the instability, to which we shall refer as a “hole.” In Figure 2 the approximate location of this region is indicated in gray. Each passage of the ensemble of trajectories past the “hole” results in the loss of some approximately constant proportion of the ensemble; these orbits leave the ensemble and soon land at the state of rest. Ensembles of trajectories that start at local sets situated near the entrance to the “hole,” tend to have shorter average lifetimes than ensembles that originate from local sets situated far from it.

**Figure 4:**
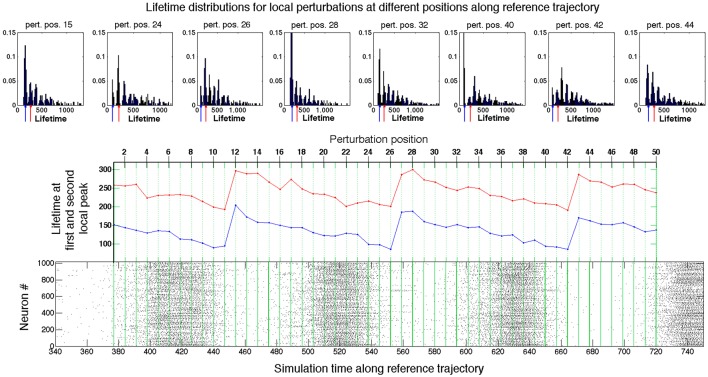
**Probing the neighborhood of the chaotic set**. Bottom panel: Raster plot of the spiking activity in the network during the motion on the reference trajectory. Green lines: positions of perturbation along the reference trajectory (see text for details). Top panel: Exemplary distributions of lifetimes at different perturbation positions. Middle panel: Dependence of the first two peaks in distributions of lifetimes on the position of perturbation along the reference trajectory. Red: first peak; blue: second peak.

Results of local investigation are graphically represented in Figure 4. The raster plot in the bottom panel shows the spiking activity of the network when the latter traverses the reference trajectory R(*t*). The green lines within the raster plot define the positions at (*t*_0_ + *k* Δ *t*) where the local perturbations were generated. The top row of Figure 4 presents examples of the lifetime distributions for local sets of “secondary” initial conditions at various perturbation positions P*_k_*. Unlike Figure 3, the distributions are non-monotonic and exhibit local maxima (peaks). The diagram in the middle of Figure 4 presents the lifetimes for the first and second local peak (respectively in blue and red) at varying perturbation positions. The latter are aligned with the raster plot at the bottom, in accordance with Equation (8).

In the raster plot, the time interval between the starts or ends of two consecutive epochs of high network activity is roughly about 100 ms; we denote it by τ. For the fixed network architecture and fixed values of parameters, the value of τ does not depend strongly on the initial conditions, and appears to be the typical time that the trajectory needs to perform one turn along the chaotic set. On the fictitious phase plane of 〈*v*〉 vs. 〈*u*〉 sketched in Figure 2, τ is the time that a system requires for one big loop. This description in terms of averaged variables, is, of course, oversimplified and rough: it ignores the complicated topology of the chaotic region in the high-dimensional phase space; nevertheless, it appears to be compatible with the observations.

The peaks in the distributions from the top row of Figure 4 confirm that in every position certain lifetimes are favored. The difference between the most probable lifetimes at the first and the second peaks delivers the familiar time value of ≈ 100 ms. The same holds for the difference between the lifetimes at any two subsequent peaks in the distributions on the top of the figure. When the perturbation position *P_j_* is shifted along the reference curve R(*t*) (middle panel, cf. also Figure 2), the values of the most probable lifetimes vary, and, despite fluctuations, display the clear tendency: gradual decrease in the peak lifetimes is followed by large jumps. Those jumps indicate the passage through subregion(s) from where trajectories tend to escape, i.e., the “hole(s).” Since the reference trajectory is sufficiently long and makes many turns in the fictitious phase plane, it repeatedly traverses the neighborhood of the “hole.” Trajectories starting from perturbed initial conditions roughly follow R(*t*), and also arrive at the “hole,” where some of them escape from the ensemble. The survivors have to accomplish a new oscillatory cycle before they arrive at the “hole” again; during this time the ensemble suffers (almost) no losses.

The closer the perturbation position is to the “hole,” the shorter is the lifetime of trajectories that escape the chaotic region at the first passage: the lifetime at the first peak. The lifetimes at the subsequent peaks are accordingly shifted by τ: the remaining trajectories have to perform another oscillation until they reach the “hole” again. When the perturbation position is moved past the “hole,” the formerly second peak becomes the first one, the third peak becomes the second one etc.; therefore, the lifetimes at the distribution peaks acquire the increment ≈ τ (cf. perturbation positions P_12_, P_27_, and P_43_ in Figure 4). The heights of different peaks within the distributions indicate the topological complexity of the chaotic region: in general, there might be more than one “hole.” For example, in the distribution corresponding to the perturbation position P_40_, the amplitude of the third peak is distinctly higher than that of the second peak. This might be due to the existence of two different “holes” with different local escape rates corresponding, e.g., to passage past two saddle points with different strengths of instability, or due to complex folding and stretching in the vicinity of the chaotic set. In any case, chaotic mixing during the motion along R(*t*) appears to be strong enough: at every passage across the “hole(s)” the prehistory of trajectories seems to play no role, and at each time the same proportion of them leaves the ensemble. Therefore, the escape rate κ, as a global characteristic of the non-attracting chaotic set, is well defined, and the joint histogram of lifetime distributions for all fifty local sets of secondary initial conditions taken together, as represented in Figure 3, displays nearly monotonic dependence close to exponential decay.

During each passage near the “hole” the ensemble loses the proportion 1–e^-κτ^≈ 0.16 of its instantaneous size. The local perturbation procedure discloses the position of the “hole” along the reference trajectory: it is always situated at the end of an epoch of high global network activity. As we discuss in the next section, at this crucial dynamical stage the activity of the network is sustained only by moderately active neurons, for which the majority of the occurring spikes do not result in spikes of their postsynaptic neurons.

#### 3.1.3. Role of modularity in maintaining the SSA

In Tomov et al. (2014) we observed that modularity favored SSA. Each module can be seen as a random network, sparsely connected to other modules. Depending on its neuronal composition, each module can sustain activity for a certain time, whereas sparse excitatory coupling enables the modules to activate each other in alternating succession. Hence, there is a probability that each module, before decaying itself to rest, (re)activates a neighboring one. This viewpoint conforms with the above phenomenological analysis, when we assume that every module possesses its own “hole.” If a module falls into its “hole” while a neighboring module is still active, there is a chance that the former will be reactivated by the latter. Hence, for activity to die out, the modules should enter their “holes” approximately simultaneously; asynchrony between the modules would sustain the activity.

**Figure 5:**
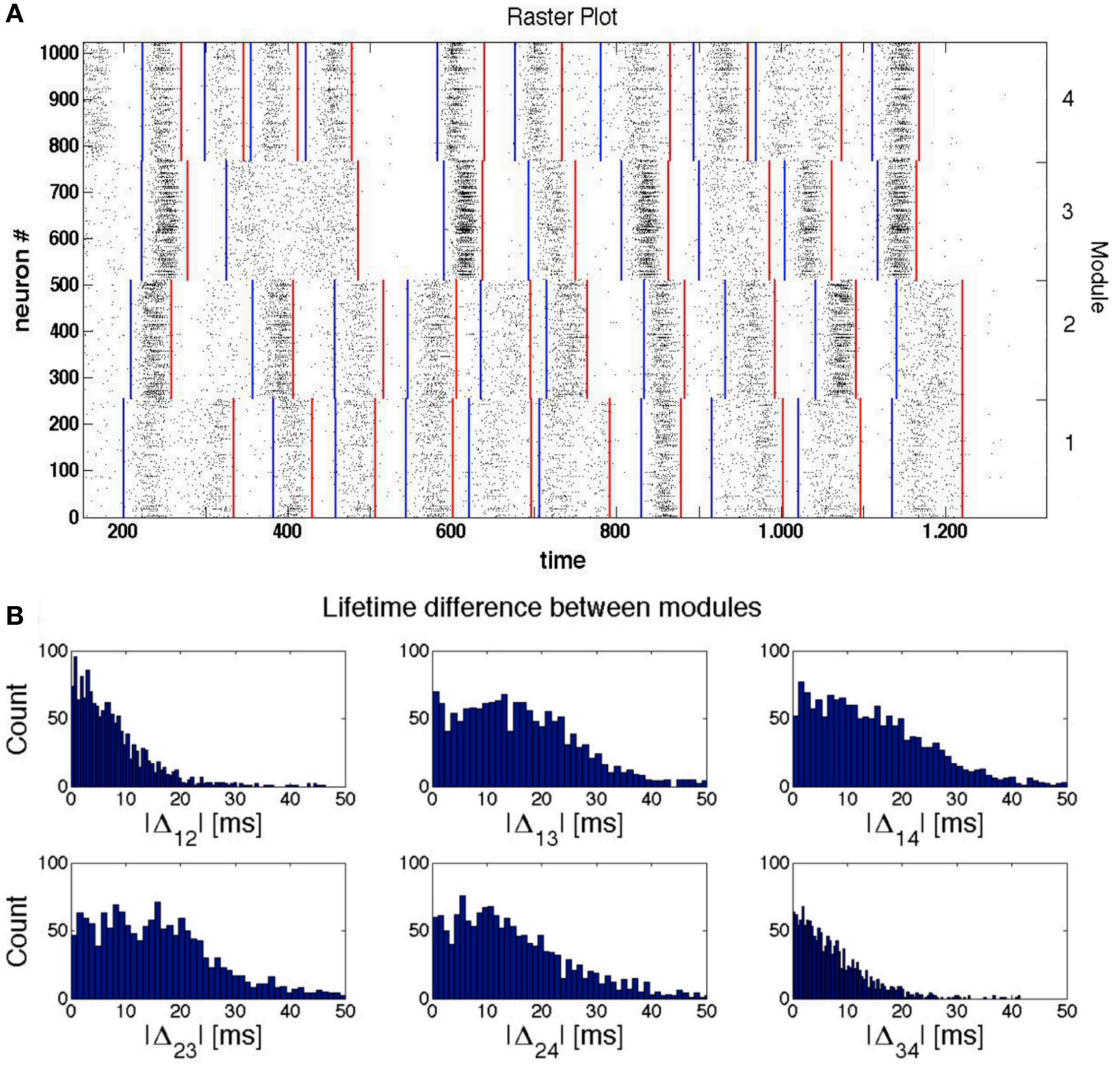
**Synchronization and temporal shifts between modules**. The network of modularity level *H* = 2 (see Figure 1), consists of LTS inhibitory neurons and RS excitatory neurons. **(A)** Sample raster plot of activity in a network. The blue and red lines indicate, respectively, the beginning and end of epochs of high network activity. **(B)** Histograms of the difference Δ*_ij_* between the ends of the last active epoch in the modules *i* and *j* (*i*, *j* = 1, 2, 3, 4) from 4 × 10^3^ trials.

This effect is illustrated by the sample raster plot in Figure 5A for a network with four modules. The blue lines indicate beginnings of epochs of high network activity *within* a module, and the red lines denote the respective ends of these epochs. By the time ≈300 the modules 3 and 4 stop firing; slightly later, the firing in them is (non-simultaneously) resumed, apparently under the influence of the still active module 1. Observing further evolution of the network we see that the SSA in the modules 3 and 4 ceases completely at *t* ≈ 500 ms for more than 100 ms, before being reactivated by signals from modules 1 and 2.

The chances of reactivation depend on the number of excitatory connections from the active module to the inactive one. While constructing the network (cf. Section 2.1 in Methods), we made a distinction between the hierarchically close and hierarchically distant modules: in the considered example, the excitatory connectivity between the close modules is nearly twice as high as between the distant ones. Hence, for hierarchically close modules the probability of mutual reactivation in the case when one of them falls into the “hole” is distinctly higher than for the modules that are hierarchically distant.

This conjecture is corroborated by Figure 5 that represents the histograms of temporal differences Δ*_ij_* between the endpoints of *the last* active epochs, respectively, in modules *i* and *j* (*i*, *j* = 1, 2, 3, 4). The histograms were computed for all pairs of modules over 2^12^ different initial conditions.

Conversely, if we compare the time differences between the moments when the modules enter their “holes” in the very last activity epoch, the probability to observe a noticeable time difference is higher for modules that are hierarchically distant. This is confirmed by the distributions of Δ_12_ and Δ_34_ from Figure 5B. In any case, however, this time difference remains small compared to the duration of the epochs of low network activity.

Summarizing, the explained mechanism of stabilization of SSA is twofold:

1. through excitatory intermodular connections, the modules are able to mutually reactivate each other, so that cessation of activity in one of them can be reversed due to the influence of the neighboring modules.
2. due to sparseness of connections between the modules, the coupling between them is too weak to induce the full synchrony. Therefore, the events (onset and decay of the active epoch) in different modules do not coincide in time. As a consequence, when activity decays in one of the modules, it is often still present in one or several of the other modules, and there are good chances that the neighbors will awake the dormant module to new activity.

In this situation, an increase of the overall connectivity would enhance the first aspect but definitely lower the second one. Higher *intermodular* connectivity can impose synchrony which will be harmful in the long run: when activity in all modules synchronously halts, nobody is left to initiate the revival.

### 3.2. SSA: single neuron perspective

#### 3.2.1. Firing patterns: chattering of non-chattering neurons

**Figure 6:**
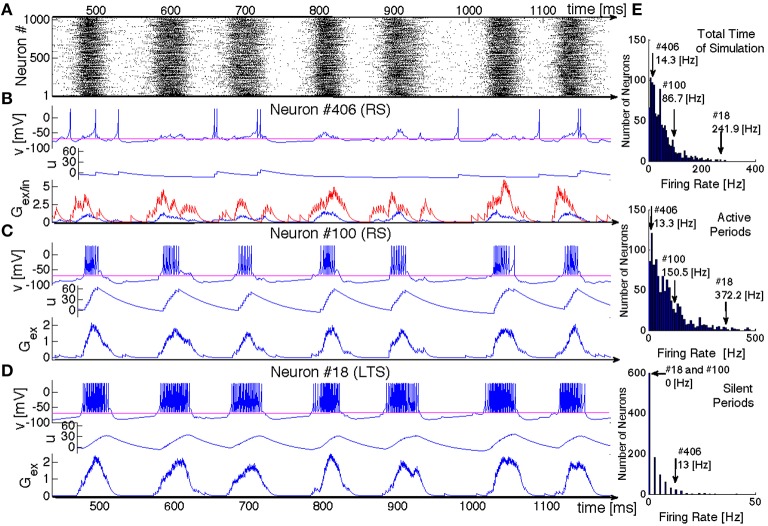
**SSA state in a network of hierarchical level *H* = 0 with LTS inhibitory neurons and a mixture of excitatory neurons: 80%RS and 20%CH**. **(A)** Raster plot: spiking activity of the network within 700 ms. **(B–D)** Evolution of variables for three exemplary neurons. Top panels: voltage *v*, middle panels: recovery variable *u*, bottom panels: synaptic conductances *G*_ex_ (in blue) and *G*_in_ (in red). Neurons # 406 and # 100 are excitatory RS. Neuron # 18 is inhibitory LTS. **(E)** Distributions of mean firing rates for all neurons in the network: for the whole duration of the SSA state (upper histogram), for the epochs of high network activity (middle histogram), and for the epochs of low network activity (bottom histogram). Positions of three neurons shown in **(B–D)** in these distributions are indicated by arrows.

Now we shall look at the inner dynamics of SSA from the point of view of its individual participants. Figure 6 presents a typical oscillatory SSA state in the network. Its top panel (Figure 6A) shows the raster plot of the system, and the panels below show time series representing the dynamical states of three sample units: two excitatory neurons and one inhibitory neuron (respectively, Figures 6B–D) aligned with the raster plot. For each of the neurons we plot the membrane potential *v*, the membrane recovery variable *u* as well as the synaptic conductances *G*_ex/in_.

Although both excitatory neurons belong to the same RS type, their behavior is remarkably different. Neuron #406 exhibits irregular spiking at low firing rate (~ 14 Hz), seemingly uncorrelated with epochs of high global network activity. This low frequency can be understood, taken into account the dominating presynaptic inhibitory input: the spikes always occur when the excitatory conductance exceeds the inhibitory (see *G*_ex/in_ diagram in Figure 6B, where the inhibitory input is represented in red and the excitatory in blue). The time series of the membrane potential *v*(*t*) confirms that during the epochs of high global network activity the neuron is in the “up” state (e.g., the range between 580 and 620 ms in Figure 6). During such epochs, global activity enhances both inhibitory and excitatory synaptic conductances, but the former is larger and, hence, for most of the time the spiking is hampered. Figure 6E shows that this neuron shares a typical firing rate of the whole ensemble and lies at the peak of the firing rate distribution: the majority of the RS neurons behaves similarly.

In contrast, neuron #100 exhibits bursting-like behavior, strongly correlated with global network activity. Its firing rate is ~ 86 Hz, if estimated over the whole length of simulation; the actual firing rate, restricted to epochs of high global network activity, reaches 150 Hz, while no spike occurs during the inactive epochs. This unit represents a more exotic subclass of RS neurons: they possess unusually high firing rates and, in fact, behave like CH neurons. Notably, within the distribution of the firing rates, neuron #100 is not placed at the very end, since that distribution includes also “genuine” CH neurons that naturally tend to have higher spiking frequencies. In the same network, this exotic behavior was also observed for LTS neurons (cf. Figure 6D); in the corresponding architectures it was found for FS and IB neurons as well. As we discuss in the next section, the chattering behavior of a non-chattering neuron is a consequence of the embedding of the neuron in the network. Remarkably, in electrophysiological experiments a regularly spiking neuron like the RS #100 might be misidentified as a chattering one.

The RS neuron #100 is, in a sense, an extreme case: it does not receive any inhibitory presynaptic input from the network and, as shown in Figure 6, always behaves like a CH neuron (we explain the origin of this behavior in the next subsection). There are, however, numerous RS neurons that receive inhibitory input but nevertheless tend to have higher firing frequencies than the typical neuron #406: they can exhibit bursting-like behavior within one epoch of high global network activity, while producing few spikes within another epoch or even completely skipping it (not shown here). Similar behavior of RS neurons naturally embedded in a network was observed empirically in Kang and Kayano (1994) and Steriade (2001) where “*work in cortical slices also showed that regular spiking neurons may develop their type of discharges into those of fast-rhythmic-bursting neurons by repeated application of depolarizing current pulses.”* Chattering behavior has also been reported for inhibitory neurons in cortical slices (Steriade et al., 1998; Steriade, 2004), contradicting the common opinion that chattering-like spiking patterns occur only in pyramidal neurons (Gray and McCormick, 1996).

A closer comparison shows that the spiking patterns of the inhibitory and the excitatory neurons, albeit qualitatively similar, bear apparent distinctions: as a rule, an epoch of high activity for the LTS neuron in the panel D starts earlier, breaks up later and is “denser” (contains more spikes) than for its RS counterpart from the panel C. As their name tells, the LTS neurons need less presynaptic excitatory input in order to spike. Hence, the lower excitatory input generated by their environments at the beginning and at the end of active epochs suffices to sustain their firing.

A sufficiently close estimate of the number of neurons that participate in all epochs of global activity can be obtained if we assume that this group consists of the neurons that, on one hand, do not receive inhibitory input and, on the other hand, receive sufficiently strong excitatory input. Recall that in our random network of *N* = 2^10^ neurons the probability for a given pair of neurons (*i*, *j*) to have an excitatory (inhibitory) connection *i* → *j* is *p_e_* = 0.008, (*p_i_* = 0.002). Then, we can expect that $N{\left(1-p_{i}\right)}^{N-1}\approx 132$ neurons get no inhibitory input. Some of these, however, do not receive sufficient excitatory input as well. The probability for a neuron to have *exactly j* excitatory presynaptic connections is

$$\frac{p_{e}^{j}{\left(1-p_{e}\right)}^{N-1-j}(N-1)!}{j!(N-1-j)!}.$$

Discarding from 132 the values with *j* ≤ 5 (a reasonable estimate for a CH neuron), we arrive at the estimate 109. For comparison: during the active epochs of the SSA that we observed in the network composed of 80% RS neurons and 20% LTS ones, the average number of frequently firing neurons was 107.

#### 3.2.2. Phase plane description for a single neuron

**Figure 7:**
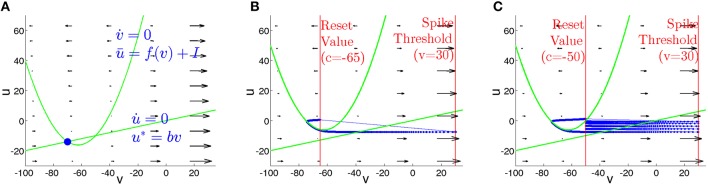
**Phase plane for isolated RS and CH neurons**. Green curves: nullclines. **(A)** *I* = 0. Nullclines intersect at states of equilibrium. Blue circle: stable equilibrium. **(B,C)** Phase portraits at *I* = 10. Thick blue curves: trajectories. Thin blue lines: resets. Vertical red lines: values of *V* at the threshold *V*_peak_ and reset *c*. **(B)** Regular spiking neuron. **(C)** Chattering neuron.

The clue to the behavior of neurons lies in the combination of their individual properties with the influence of the network. We begin the description with the case of the isolated neuron under constant input current *I*. On the phase plane, the dynamics of the system (2) is constrained by the location of two nullclines. The nullcline of voltage *v*,

(10) $$u=\bar{u}=\text{f}(v)+I$$

is a quadratic parabola separating the phase plane region with $\dot{v}<0$ (above *ū*) from the region with $\dot{v}>0$ (below *ū*). The nullcline of the membrane recovery variable *u*

(11) $$u=u^{*}=bv$$

is a straight line. Figure 7 sketches typical phase portraits of regular spiking and chattering neurons. Location of the nullcline *ū* is independent of the parameters (*a*, *b*, *c*, *d*); variation of the input current *I* shifts *ū* in the vertical direction. The nullcline u^*^ depends on the parameter *b* that controls its slope and therefore determines, at *I* = 0, the resting state of the neuron. Since the neurons of the RS and CH types share the same value of *b*, their phase portraits at *I* = 0 are identical. For zero or sufficiently small input *I* the nullclines intersect at two points (Figure 7A): the stable (left) and unstable (right) equilibrium. At *I* = (25*b*^2^ − 250*b* + 65)/4 these equilibria disappear in a saddle node bifurcation, and at higher current values the neuron spikes. Figures 7B,C show phase portraits of the RS and CH neurons, respectively, at *I* = 10. Distinctions between the two types proceed on the one hand, from different (controlled by parameter *d*) increments of the variable *u* after each spike, that are considerably higher for the RS neuron and, on the other hand, from difference in the reset value *c*. The effect is clearly seen in the plots: the RS neuron is instantaneously reset to a position above the parabola, whereas the CH neuron performs several *cycles* (spikes) until the value of *u* becomes sufficiently large to exceed the nullcline *ū*.

The timescale separation between *v* and *u*, caused by the small parameter *a* in Equation (2), is visualized in the plot of the vector field on the phase plane: during the spiking stage, *v* rapidly changes, whereas the value of *u* remains approximately constant. The two time scales are comparable only during the visits of the trajectory in the vicinity of the nullcline *ū*, where the velocity $\dot{v}$ becomes small.

The dynamical properties of the neurons are affected by their embedding into a network. The formal assignment of neuronal class at *each site* is based on the characteristics from Izhikevich (2003), where for every neuron type the parameters are gauged with respect to a constant presynaptic input current. That picture can change when the neuron becomes part of a complex network, where the input current is subject to synaptic interactions and is therefore time-dependent. In accordance to (10), the location of the nullcline *ū* on the phase plane explicitely depends on *I*(*t*), whereas u^*^ remains fixed. As a result, the formerly constant phase portrait, constraining the behavior of the given neuron and rendering its type, varies in time, and the neuron behaves according to its environment.

**Figure 8:**
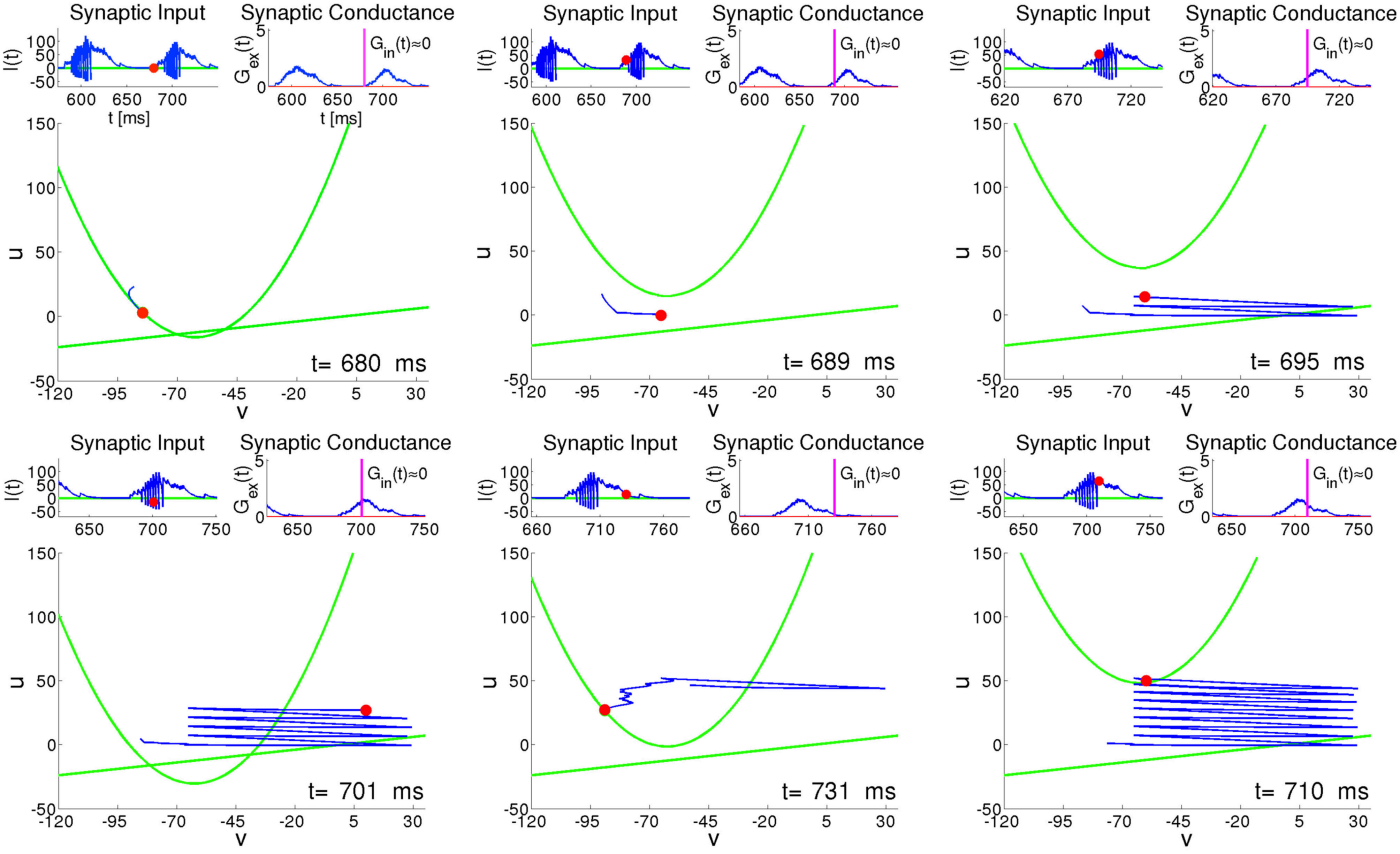
**RS neuron embedded in the network: snapshots of the phase plane at different instants of time**. Green curves: nullclines of Equation (2); Red circle: instant state of the neuron. Blue curve: recent trajectory of the neuron. Left upper inset: presynaptic input current *I*(*t*). Red circle: instantaneous value of current. Right upper inset: synaptic conductance *G*(*t*). Vertical line: instantaneous value of conductance.

Figure 8 presents six characteristic snapshots of the phase portrait for the RS neuron #100 from Figure 6. Each snapshot shows the instantaneous state, along with the recent trajectory and the two nullclines. Insets above each snapshot display the presynaptic input current *I*(*t*) and the synaptic conductance *G*(*t*). We concentrate on the time interval around one of the epochs of high network activity lasting from *t* ≈ 680 to *t* ≈ 740 (see Figure 6A). During the preceding period of low network activity between *t* ≈ 630 and *t* ≈ 680, the synaptic input of the RS neuron #100 is practically absent, and the trajectory relaxes along the parabolic nullcline toward the state of rest (Figure 8, *t* = 680); during this relaxation both $\dot{v}\left(t\right)$ and $\dot{v}\left(t\right)$ stay small, with comparable timescales. At the beginning of the active period the excitatory synaptic input shifts the parabolic nullcline upwards, and the neuron is free to spike (Figure 8, *t* = 689). In the next two plots (Figure 8, *t* = 695 and Figure 8, *t* = 701) we see that while *G*_ex_(*t*) is fluctuating weakly, *I*(*t*) exhibits strong rapid variation caused both by fast evolution of the variable *v* and by its reset after each spike. During this active phase, oscillations of the current *I*(*t*) provoke large movements of the parabolic nullcline [see Equation (10) and Equation (4)]. A growth of the synaptic input due to the increasing activity of the network provides an additional shift of the nullcline *ū* upwards. Unlike the case of constant input current in Figure 7, this shift ensures that after each spike the reset of the recovery variable *u*↦*u* + *d* leaves the system below *ū*, allowing for another spike. This explains why the RS neuron tends to behave like a CH neuron during the active epochs (see Figure 8, *t* = 701 and *t* = 710). Similar bursting behavior is typical for all highly active neurons, regardless of their electrophysiological class. The bursting-like process endures either until the neuron, after a spike, is instantaneously reset above the nullcline *ū*, or until its trajectory (*v*(*t*), *u*(*t*)) is trapped by *ū* (see Figure 8, *t* = 710). Subsequently, the neuron enters relaxational evolution with small $\dot{v}$, slowly moving toward the state of rest (see Figure 8, *t* = 731).

Since the membrane potential *v* is directly available in electrophysiological experiments, most computational studies understandably concentrate on the evolution of *v*. The above argumentation, however, leaves the decisive role in network dynamics to the membrane recovery variable *u*. We discuss this aspect in the next section.

#### 3.2.3. Different stages of the SSA process

**Figure 9:**
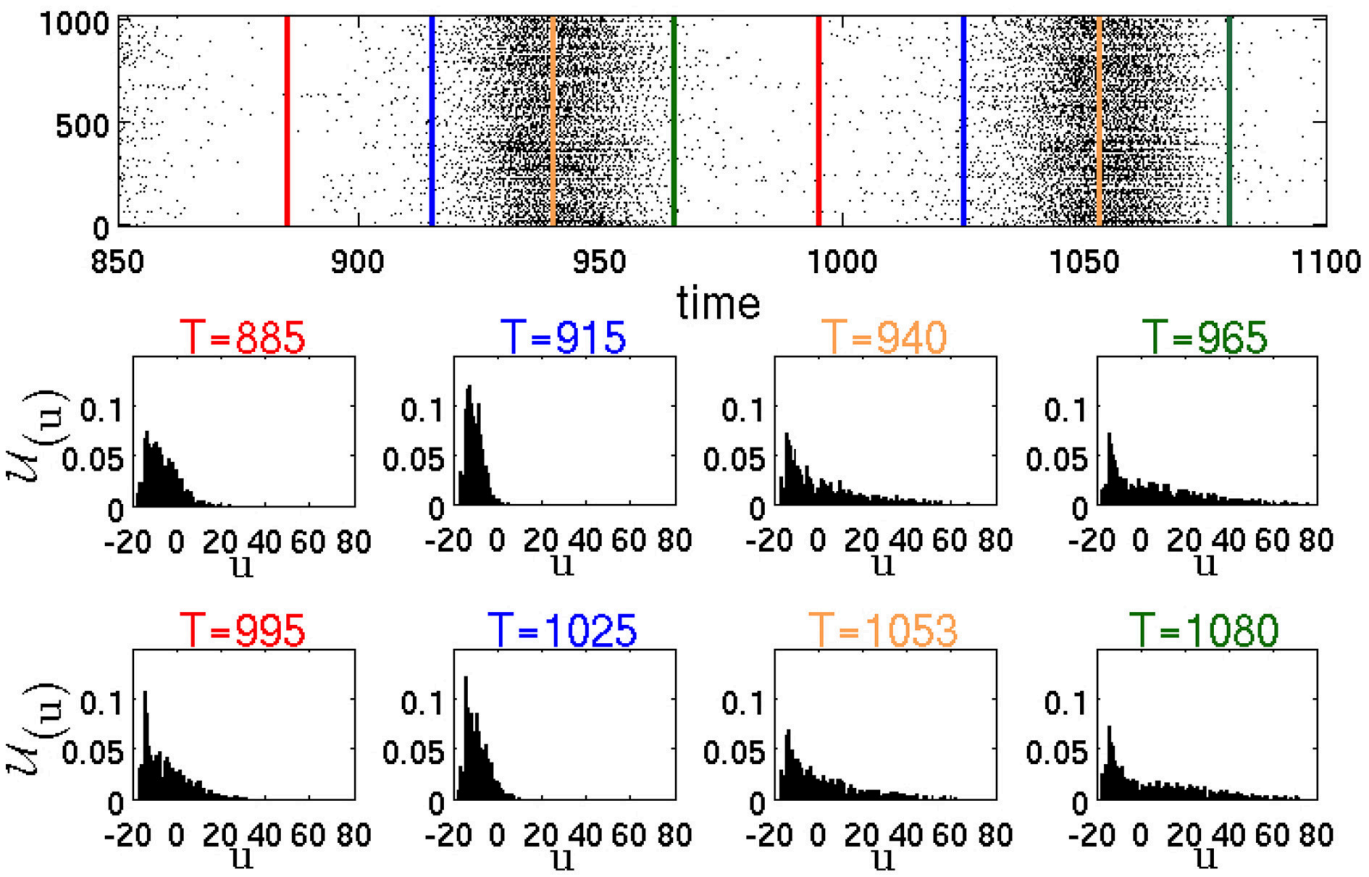
**Raster plot and temporal evolution of distribution U(*u*, δ*u*; *T*), calculated according to Equation (1) with δ*u* = 1**. Red lines in the raster plot denote midpoints of intervals between two active epochs. Blue, yellow and green lines show, respectively, the beginning, the middle and the end of active epochs.

Figure 9 represents the distribution U(*u*, δ*u*; *t*) of the membrane recovery variables *u* in the whole network at different time instants *T*. The raster plot in the figure corresponds to a simulation of the same network as in Figure 6, resulting in a typical SSA state. The instants *T* are chosen, respectively, between two active epochs or at the beginning, in the middle and at the end of an active epoch.

#### 3.2.4. Mechanism of breakdown of global activity

The instantaneous distributions, computed amid (*T* = 940, *T* = 1053) and at the end (*T* = 965, *T* = 1080) of global activity confirm, as expected, that during an active epoch the proportion of neurons with high values of *u* increases. Let us single out a highly active neuron *h*, like the RS neuron #100 from Figure 6. At the peak of an active epoch all its presynaptic neurons (in this case all of them are excitatory) are highly active and, similarly to *h*, fire at an approximately constant rate. The total synaptic input into *h* reaches a *maximal* level that remains roughly constant throughout the epoch. Due to this temporary saturation of the input, the nullcline *ū* of *h* acquires its highest position and does not move upwards anymore. Meanwhile, the membrane recovery variable *u* of *h* gets incremented by *d* after each spike; this eventually puts the reset system beyond *ū*, halting thereby the bursting regime. The inability of the neuron *h* to fire a next spike subsequently shifts downwards the nullclines *ū* of all its postsynaptic neighbors. Its presynaptic neighbors experience eventually the same, and the nullcline of *h* starts lowering as well. A potential new presynaptic excitatory input may once again shift the nullcline *ū* upwards, leading to a small positive $\dot{v}$ that may or may not result in a new spike (see the jitter in the end segment of the trajectory in Figure 8 *t* = 731). Overall, this process results in a definite drop in the spiking rate of the whole network, leading to lowering of the nullclines *ū* of most neurons, that, in its turn, slows their spiking frequencies further. Eventually, all most active neurons get trapped by their parabolic nullclines and begin relaxation toward their resting states. This marks the end of the active epoch.

#### 3.2.5. Role of inhibition in resumption of activity

Noteworthy, breakdown of activity is not directly influenced by inhibition. In fact, for example, at (*g*_ex,_*g*_in_) = (0.15, 0) (this effectively ensures closure of all inhibitory synapses), in the presence of ongoing external stimulus the network displays global activity oscillations. However, as discussed in Tomov et al. (2014), in the absence of inhibition, such activity states can not be sustained after elimination of the external stimulus. Although inhibition does not cause the end of an epoch of high global activity, it is pivotal for the onset of new active epochs. This becomes clear from the distributions U(*u*, δ*u*; *t*). According to the distributions at *T* = 965 and *T* = 1080, by the end of the active epochs there still remain the neurons with low *u* values. We call them moderately active neurons. The peak of the distributions is close to the values of *u* for the resting states. Most of the moderately active neurons are postsynaptic to formerly active inhibitory neurons, the latter being at the stage of slow relaxation. At the end of the active epoch, the moderately active neurons receive less presynaptic inhibition and, being near their resting state, do not need much excitation in order to spike. Remark that at this stage a large number of neurons postsynaptic to the moderately active neurons are relaxing, almost insensitive to the input, and, therefore, most of the newly produced spikes do not provoke firing of the postsynaptic neurons (as in the case of the jitter of RS neuron #100 in Figure 8 *t* = 731). The bulk of the newly produced network activity gets absorbed by the relaxing neurons. At this stage, the activity of the network is sustained only within the “pool” of moderately active neurons. In terms of the phenomenological description from Section 3.1, this stage corresponds to the passage through the “hole.” Note the importance of inhibition: if during the highly active periods it is insufficient, there will be less moderately active neurons at the end of the active epochs which will decrease the probability of sustaining the activity. In the distributions in Figure 9 at *T* = 885 and *T* = 995 between the active epochs, the tails are shifted from high toward low *u* values. This shift corresponds to the arrival of formerly active neurons at their resting states. As a result, the number of neurons that are able to contribute to the network activity is growing. At the beginning of the active epochs (*T* = 915, *T* = 1025), the distributions are concentrated at low *u* values close to the resting states, that marks the end of the relaxation. At this stage the network becomes highly excitable. Here, most neurons possess low *u* values and, therefore, newly produced spikes lead with a higher probability to subsequent spikes in the postsynaptic neurons.

#### 3.2.6. Peculiarities of different types of neurons

**Figure 10:**
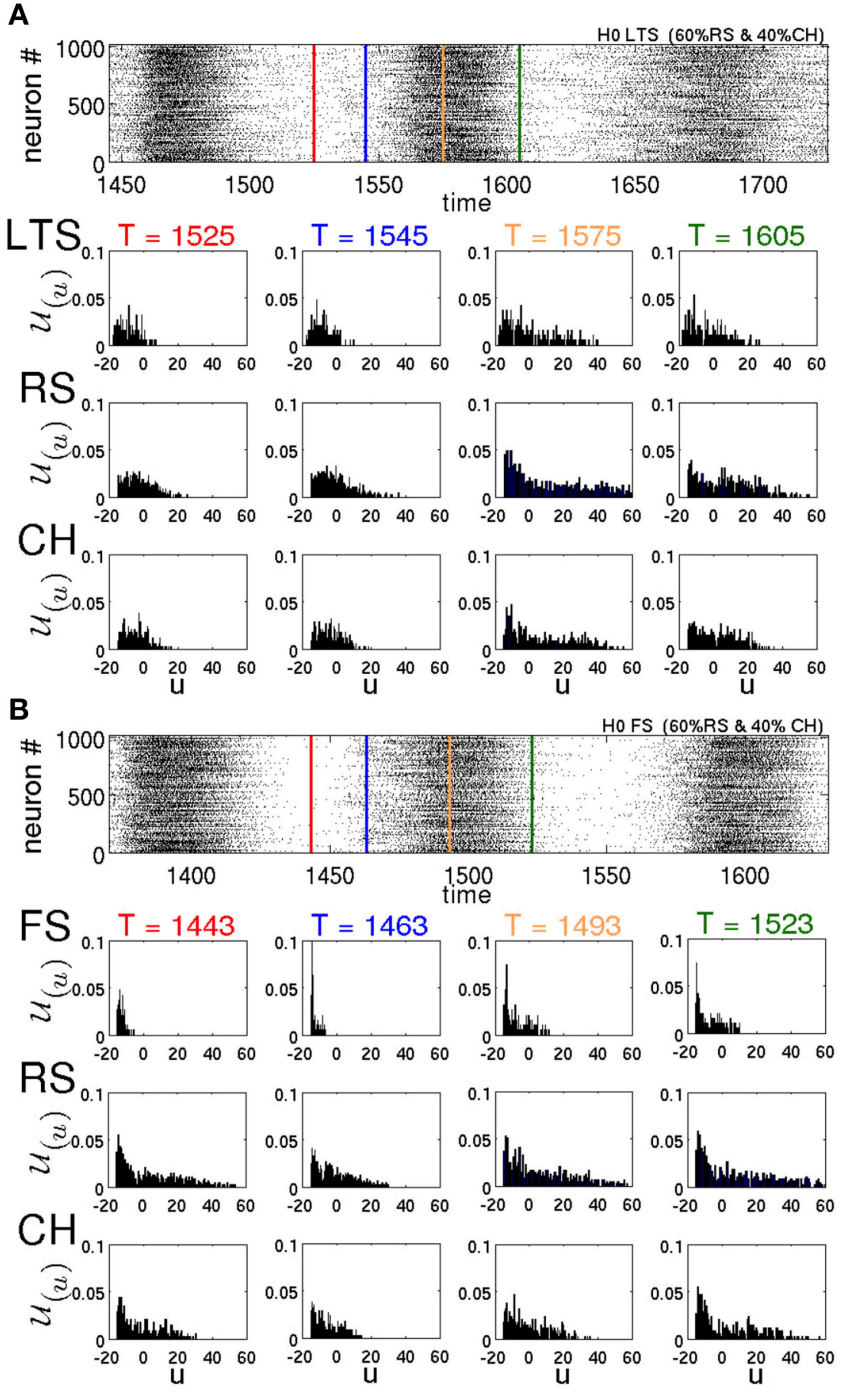
**Raster plots and distributions of recovery variable for various neuronal types in two different networks with *H* = 0**. U(*u*, δ*u*; *T*) is calculated according to Equation (1) with δ*u* = 1. In both networks 60% of excitatory neurons are of the RS type and 40% are of the CH type. **(A)** All inhibitory neurons are of the LTS type. **(B)** All inhibitory neurons are of the FS type. In the raster plots, red lines denote midpoints of intervals between two active epochs. Blue, yellow and green lines show, respectively, the beginning, the middle and the end of active epochs.

These observations help us understand the effect that different types of neurons exert on the lifetimes of oscillatory SSA. Figure 10 represents the distributions U of the variable *u* for different types of neurons at different times *T* for two different networks in typical SSA states. Both networks are random, without modularity, with 60% of all excitatory neurons being of the RS type and 40% of the CH type. In Figure 10A the inhibitory neurons are of the LTS type, whereas in Figure 10B they are of the FS type. Compare the LTS distributions in the middle (*T* = 1575 ms) and at the end (*T* = 1605 ms) of the active epoch in Figure 10A, with the corresponding FS distributions at *T* = 1493 ms and *T* = 1523 ms in Figure 10B. Notably, the LTS neurons tend to possess higher *u* values than the FS ones. Since the increase *d* of the recovery variables for the LTS and FS neurons is the same (see Table 1), intuitively it can be expected that the difference is due to a higher firing rate of the LTS neurons during the active epochs. However, this is not the case: as discussed in Tomov et al. (2014), maximal firing rates of the FS neurons are consistently higher than those of the LTS neurons. The difference in the distributions is due to the dynamical properties of the LTS and FS neurons. The parameter *a* that governs the timescale of the membrane recovery variable *u* [see Equation (2)] is five times larger for an FS neuron than for other types (cf. Table 1); hence, during the slow relaxation along the parabolic nullcline after a spiking process, FS neurons recover about ten times faster than the other neuron types. Besides, the FS and the LTS neurons possess different values of the parameter b that also influences the dynamics of the recovery variable *u* (see Table 1). Therefore, a difference in distributions U(u) between these neuron types is present all through the SSA process. This circumstance becomes crucial after the breakdown of every epoch of high global network activity, when the previously active neurons are slowly relaxing, and the network activity is sustained by the “pool” of moderately active neurons. In this state, the higher value of *a* and the lower values of *u* cause the FS neurons to recover much faster, compared to the LTS neurons. Notably, between the active epochs (red lines in the raster plots) at *T* = 1525 ms for LTS neurons and at *T* = 1443 ms for FS neurons, respectively, the distribution of the *u* variables of the FS neurons is shifted toward lower values of the membrane recovery variable. This shift is still recognizable in the beginning of active epochs (blue lines in the raster plots), at *T* = 1554 ms for LTS neurons and at *T* = 1463 ms for FS neurons, respectively. The FS neurons recover not only faster than the LTS, but much faster than excitatory neurons as well. As a consequence, they become excitable earlier than the excitatory neurons and inhibit the “pool” of moderately active neurons during the state of low network activity, diminishing the probability for sustaining the activity. Therefore, the mean spiking frequency in the epochs of low network activity is much higher for a network comprising LTS inhibitory neurons than for a network with FS neurons. This is clearly visible in the raster plots in Figures 10A,B, respectively.

In Tomov et al. (2014) we observed that the probability of finding long-living SSA is higher in networks that included not only the RS excitatory neurons, but the CH neurons as well. This can be explained following the same reasoning in terms of individual variables of the neurons. One of the differences between CH and RS neurons is in the value *c* to which the voltage *v* is reset after a spike. For the CH neurons, *c* is higher, and this, under time-dependent input, increases the chances for subsequent spikes (bursts). Besides, there is also a difference in the increment *d* that the membrane recovery variable *u* acquires at the reset. For the CH neurons, *d* is four times smaller than for the RS neurons, therefore, during the epochs of high network activity the variables *u* of the CH neurons grow slower than for the RS neurons. Hence, CH neurons tend to possess lower values of *u*, compared to RS neurons; see Figure 10. Accordingly, at the end of an epoch of high network activity, the membrane recovery variable of CH neurons reaches low values easier, causing these neurons to contribute to the “pool” of moderately active neurons and hence increasing the probability of sustaining the activity during the low-activity epoch.

## 4. Discussion

We have characterized the local and global mechanisms, responsible for the existence of self-sustained activity in a hierarchical modular network composed of mixtures of cells of different electrophysiological classes found in the cortex. The model that we extensively studied and described previously (Tomov et al., 2014), displays oscillatory SSA states akin to up and down states found in cortical tissue with lifetimes that depend on the specific neuronal mixture and on the number of modules in the network. Specifically, networks with excitatory cells of the RS and CH types and inhibitory cells of the LTS type have higher probability of displaying long-lived SSA than networks comprised of other neuronal mixtures, and this probability is enhanced when the number of modules in the network is increased.

To interpret the behavior in the high-dimensional phase space of the model, we used the phenomenological idea of a relatively small and confined “hole” through which trajectories may escape from the chaotic set during their evolution. This allowed us to explain qualitatively the global oscillations in the network and their unpredictable breakdowns at the ends of the high activity phases, as well as the exponential distributions of the SSA lifetimes. Reasoning along the same lines, we explained the facilitating effect of modularity upon SSA. Hierarchically distant modules tend to have higher degrees of asynchrony during their activities, and therefore tend to fall into their “holes” at different times. Hence, a rise in the hierarchical level of the network, by increasing the number of modules, enhances intermodular asynchrony and the likelihood of maintaining the SSA. Important for the effectiveness of the modularity effect is the sparseness of intermodular connectivity.

Proceeding from global phase space of the whole network to local dynamics of its units, we have interrelated the breakdown of global activity at the end of high activity epochs and the dynamics of the time-dependent nullcline of the voltage variable of the single neuron. In this situation, characterization in terms of voltage alone is insufficient: the breakdown is forecasted by the shift upwards in the instantaneous distribution of the membrane recovery variable.

The important finding of our analysis concerns the role of inhibition: not especially relevant for the global activity breakdown, it is crucial in the preparation and start of each new high activity epoch. Relying on the intrinsic dynamics, we succeed in explaining, why networks with inhibitory LTS neurons tend to display longer lifetimes than those with inhibitory FS neurons: the former are more efficient in restarting activity epochs than the latter.

Other important finding from our single-neuron phase plane analysis is the remarkable role of truly bursting (chattering) neurons in the network. Their presence increases the likelihood that the network survives a low activity epoch and passes to a new epoch of high activity. This explains our previous finding that the addition of CH neurons to the network contributes to long-lived SSA.

Based on our analysis, we can make the following predictions, that might be tested in *in vivo* cortical preparations: If it is possible to selectively silence or block synaptic activity of inhibitory LTS neurons, while keeping inhibitory FS neurons functioning normally, the probability of observing long-lived self-sustained oscillatory activity would be drastically reduced. Similarly, if it is possible to silence or block synaptic activity of CH neurons, the probability of observing long-lived oscillatory SSA would be reduced as well.

Remarkably, our simulations have shown that some neurons with parameters tuned to reproduce regular spiking behavior when stimulated by constant input current, change to a bursting-like (chattering) firing pattern when embedded in the network. Similar effect has been detected for inhibitory neurons, tuned to produce LTS and FS firing patterns when in isolation. This confirms that the network activity can influence and qualitatively change the spiking pattern of a neuron model, although its parameters stay fixed throughout the simulation. Changes in the qualitative spiking profile of neurons due to the background activity of the network have been observed experimentally, both *in vitro* and *in vivo* (Kang and Kayano, 1994; Steriade et al., 1998; Steriade, 2001; Shu et al., 2003; Steriade, 2004; Altwegg-Boussac et al., 2014), and also have been suggested by other theoretical models (Hô and Destexhe, 2000). Our simulations of the cortical network deliver an unambiguous illustration of this effect.

**Figure 11:**
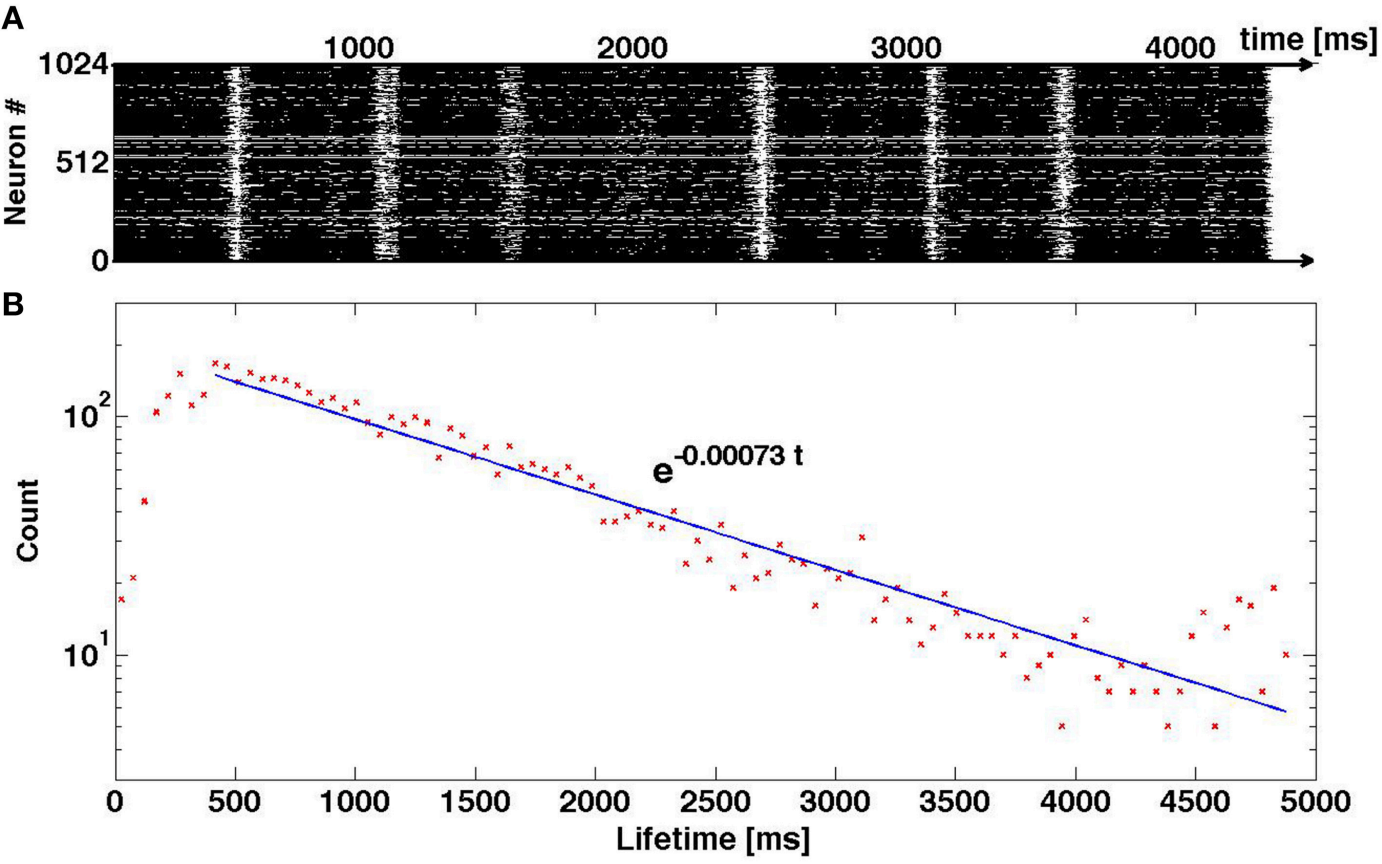
**Transiently chaotic oscillatory SSA in the network of AdEx neurons**. **(A)** Raster plot of activity. **(B)** Distribution of lifetimes in the ensemble of 5 × 10^3^ trajectories where initial conditions were created by varying the proportion of randomly chosen stimulated neurons: 1, 1/2, 1/4; the amplitude of the external current from *I_stim_* = 500 to *I_stim_* = 800; and the duration of stimulation from 100 to 500 ms.

A natural question is: How much of these results is dependent on the particular neuron model? Our interpretation of the oscillatory pattern of SSA, with alternating high and low activity epochs, is based on the voltage and recovery variables of single neurons, and their interaction within the coupled system of differential equations (2). In Figure 11 we present preliminary results of numerical simulations for a network that has the same structure and same density of synaptic connections as above, but is ruled by a different neuronal model: it is the adaptive exponential integrate-and-fire (AdEx) model (Brette and Gerstner, 2005). The difference between Equation (2) and the AdEx Equation (6) is, essentially, the difference between the quadratic and the exponential functions in the equation for *dV*/*dt*. Like in the case of Equation (2), the AdEx neurons have been originally stimulated by external current within a certain time interval; this procedure created initial conditions for the subsequent free evolution. When the parameters of the AdEx neurons are properly tuned (see values in Section 2.3), the familiar pattern of transient self-sustained oscillations is observed: irregular alternation of epochs of high and low global activity, followed by the abrupt decay to the state of rest (top panel of Figure 11). Here, as well, lifetime of self-sustained activity turns out to be rather sensitive to variations of the preparation protocol: minor changes in proportions of stimulated neurons, strength and duration of external stimulation result in big—and seemingly unpredictable—changes in the total duration of activity until the final decay. The bottom panel of Figure 11 testifies that in the sufficiently large ensemble of trajectories the distribution of lifetimes is unambiguously exponential. This allows us to conjecture that what matters for transiently chaotic oscillatory SSA is the qualitative shape of the nullclines, and not so much the exact form of equations.

The role of modularity is not crucial, but it seems to be less sensitive to the particularities of the neuron model. Therefore, going beyond the particular case, we conclude that the generic requirements for an oscillatory behavior like the one observed are: a network of excitatory and inhibitory neurons with a balance between excitation and inhibition, and some form of build-up and saturation mechanism for the spiking activity (as present in both Izhikevich and AdEx models), to promote activity breakdown. The significant role of the inhibitory neurons would be to leave a residual spiking neuronal activity after breakup, in order to allow for the ignition of the next epoch of high activity.

## Author contributions

PT, RP, AR, and MZ conceived the work, analyzed the results and wrote the manuscript. PT and RP developed the codes and performed the computations.

### Conflict of interest statement

The authors declare that the research was conducted in the absence of any commercial or financial relationships that could be construed as a potential conflict of interest.
